# Identification of *Plasmodium falciparum* specific translation inhibitors from the MMV Malaria Box using a high throughput in vitro translation screen

**DOI:** 10.1186/s12936-016-1231-8

**Published:** 2016-03-17

**Authors:** Vida Ahyong, Christine M. Sheridan, Kristoffer E. Leon, Jessica N. Witchley, Jonathan Diep, Joseph L. DeRisi

**Affiliations:** Department of Biochemistry and Biophysics, University of California San Francisco, San Francisco, CA USA; Department of Microbiology and Immunology, University of California San Francisco, San Francisco, CA USA; Howard Hughes Medical Institute, Chevy Chase, MD USA

**Keywords:** Translation, Ribosome, Anti-malarials, Screen, *Plasmodium falciparum*, MMV, Malaria Box

## Abstract

**Background:**

A major goal in the search for new anti-malarial compounds is to identify new mechanisms of action or new molecular targets. While cell-based, growth inhibition-based screening have enjoyed tremendous success, an alternative approach is to specifically assay a given pathway or essential cellular process.

**Methods:**

Here, this work describes the development of a plate-based, in vitro luciferase assay to probe for inhibitors specific to protein synthesis in *Plasmodium falciparum* through the use of an in vitro translation system derived from the parasite.

**Results:**

Using the Medicines for Malaria Venture’s Malaria Box as a pilot, 400 bioactive compounds with minimal human cytotoxicity profiles were screened, identifying eight compounds that displayed greater potency against the *P. falciparum* translation machinery relative to a mammalian translation system. Dose–response curves were determined in both translation systems to further characterize the top hit compound (MMV008270).

**Conclusions:**

This assay will be useful not only in future anti-malarial screening efforts but also in the investigation of *P. falciparum* protein synthesis and essential processes in *P. falciparum* biology.

**Electronic supplementary material:**

The online version of this article (doi:10.1186/s12936-016-1231-8) contains supplementary material, which is available to authorized users.

## Background

Identifying new anti-malarials with novel mechanisms of action is a key goal in the fight to eradicate malaria worldwide [1]. A common and very successful strategy relies on screening in vitro cultures of *Plasmodium falciparum* against large compound collections and assaying for growth inhibition in a ‘top-down’ approach to drug discovery. Using this type of approach can result in the subsequent identification of drug targets by selection of resistant strains and whole genome sequencing of these resistant strains to identify mutations (i.e., single nucleotide polymorphisms, copy number variants, insertions/deletions) that confer resistance [2–4]. However, one challenge with this approach is the high likelihood of encountering new compounds associated with targets that have previously been exploited as opposed to identifying new mechanisms of action. This has certainly been the case in recent instances, in which multiple groups have identified diverse chemical compounds that all have the same molecular determinants of resistance, such as PfATP4, and quite possibly the same mechanism of action [2, 5, 6] .

The complementary strategy is to narrow the search criteria by assaying for activity against a specific biological function or pathway. For example, this approach was used to identify a specific inhibitor of PfIspD, an enzyme essential for isoprenoid synthesis, by counter screening with growth media supplemented with isopentenyl pyrophosphate (IPP), thus narrowing hits to only those active against apicoplast targets (such as isoprenoid enzymes) [4]. Facilitating these efforts, the freely available Medicines for Malaria Venture (MMV) Malaria Box has been a welcome resource, providing biologically active compounds with unknown targets and mechanisms of action [7]. The library contains 400 chemically diverse compounds that are commercially available and pre-screened for activity in the blood stages of *P. falciparum* with minimal human cytotoxicity.

Among the possible pathways that can be functionally assayed, protein synthesis represents an attractive target, given its absolutely essential nature. Indeed, despite the fact that *P. falciparum* is a eukaryotic organism, there are ample differences between the *P. falciparum* and mammalian ribosomes that could be plausibly exploited [8, 9]. In fact, precedence for this type of inhibition of protein synthesis was exemplified in the discovery of the sordarin class of natural products which selectively inhibits fungal protein synthesis by inhibiting the yeast eukaryotic elongation factor 2 [10]. In a similar manner, a potent new compound, DDD107498, was reported to specifically inhibit *P. falciparum* protein synthesis by blocking activity of the *P. falciparum* translation eukaryotic elongation factor 2 [11]. Here, this study reports the use of a *P. falciparum* in vitro translation assay, amenable to plate-based screening, to identify inhibitors of *P. falciparum* translation present in the Malaria Box.

## Methods

### *Plasmodium falciparum* culturing

W2 strains were maintained in HYPERFlasks (Corning, Corning, NY, USA) in 500 mL RPMIc [RPMI 1640 media supplemented with 0.25 % Albumax II (GIBCO, Grand Island, NY, USA), 2 g/L sodium bicarbonate, 25 mM HEPES (pH 7.4), 0.1 mM hypoxanthine, and 50 ug/L gentamycin] in a 37 °C, 5 % O2, 5 % CO_2_ incubator in 2 % haematocrit (HC). Cells were synchronized with 5 % sorbitol treatment for two generations to achieve high synchronicity.

### Harvesting cell pellets

One liter parasite cultures grown in two 500 mL HYPER flasks were harvested in the late trophozoite stage at approximately 15 % parasitaemia by centrifugation for 5 min at 1500×*g* at room temperature and 0.06 % final saponin in Buffer A (20 mM HEPES pH 8.0, 2 mM Mg(OAc)2, 120 mM KOAc). Saponin lysed pellets were centrifuged at 4 °C 10,000×*g* for 10 min and washed once with ice-cold Buffer A. The pellet was resuspended in 2 mL of Buffer B2 [20 mM HEPES pH 8.0, 100 mM KOAc, 0.75 mM Mg(OAC)2, 2 mM DTT, 20 % glycerol, 1× protease inhibitor cocktail (Roche)], flash frozen, and stored in −80 °C freezer until the sample was ready to homogenize.

### Homogenization of cell pellets

Frozen pellets were thawed on ice and added to a 3-mL luer lock syringe, locked onto a pre-chilled cell homogenizer (Isobiotec, Germany) on ice and passed between two syringes 20 times. Lysate was centrifuged at 4 °C 16,000×*g* for 10 min and the supernatant was stored at −80 °C.

### In vitro translation assay

*Plasmodium* in vitro translation reactions were carried out v-bottom 96-well PCR plates (USA Scientific, Ocala, FL, USA) and sealed with adhesive aluminum foil plate seals (Beckman Coulter, Indianapolis, IN, USA) with the following components in 20 μL: 16 μL lysate, 1 μg T7 transcribed firefly luciferase mRNA, 10 µM amino acid mixture, 20 mM HEPES/KOH pH 8.0, 75 mM KOAc, 2 mM Mg(OAc)2, 2 mM DTT, 0.5 mM ATP, 0.1 mM GTP, 20 mM creatine phosphate, 0.2 μg/μl creatine kinase for 0.5–1.5 h at 37 °C. All liquid dispensing was performed using Rainin E4 12-channel electronic pipettes (Rainin Instruments, Oakland, CA, USA). After incubation, the reactions were quenched with a final concentration of 5 µM cycloheximide. Samples were transferred to a 96-well LUMITRAC 200 white immunology plate (Greiner Bio-One, Monroe, NC, USA). Reactions were assayed using the Promega GloMax-Multi + microplate reader with a three-second delay and ten-second integration after addition of 100 μL luciferin reagent (20 mM Tricine, 2.67 mM MgSO_4_×7H_2_O, 0.1 mM EDTA, 33.3 mM DTT, 530 μM ATP, 270 μM Acetyl CoEnzyme A, 1 mM D-Luciferin, 265 μM Magnesium Carbonate Hydroxide, pH 8.15).

Rabbit reticulocyte (Retic Lysate IVT Kit, Thermo Fisher Scientific, Waltham, MA, USA) in vitro translation assays were carried out with the following components in 20 μL: 0.5 μL 20× translation mix minus methionine, 0.5 μL 20× translation mix minus leucine, 1 μg T7 transcribed firefly luciferase mRNA, and 18 μL reticulocyte lysate for 5–20 min at 37 °C. After incubation, the reactions were quenched with 5 µM cycloheximide and assayed in the same manner as the *Plasmodium* lysates.

### Drug screening

The open-access Malaria Box was obtained from MMV. Initial screens were performed at 1 μM final drug concentration in v-bottom 96-well PCR plates (USA Scientific, Ocala, FL, USA) and sealed with adhesive aluminum foil plate seals (Beckman Coulter, Indianapolis, IN, USA). The 20 μL reaction contained the following components in 20 μL: 14 μL lysate, 1 μg T7 transcribed firefly luciferase mRNA, 10 µM amino acid mixture, 20 mM HEPES/KOH pH 8.0, 75 mM KOAc, 2 mM Mg(OAc)2, 2 mM DTT, 0.5 mM ATP, 0.1 mM GTP, 20 mM creatine phosphate, 0.2 μg/μl creatine kinase. After the 1.5 h incubation, samples were immediately quenched using 2μL of 50 μM cycloheximide stop solution and then transferred to the Lumitrac assay plate using 12-channel electronic pipettes and assayed in the same manner described in the above in vitro translation assay. IC_50_ values were determined with a constant 2.5 % DMSO, using a 12-point 1:3 titration starting at 250 μM. Data were normalized to background and DMSO-only controls.

## Results

### Development of a high-throughput, malaria-specific, in vitro translation assay

**Fig. 1:**
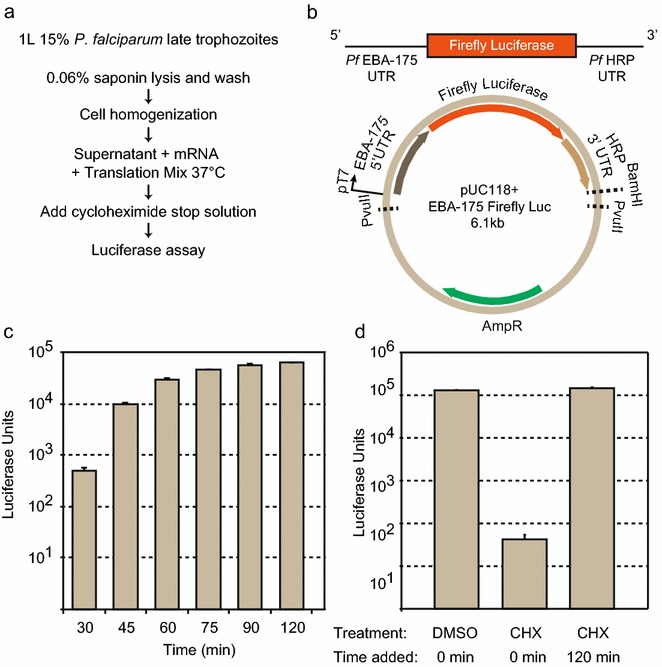
Development of a luciferase based in vitro translation assay in *Plasmodium falciparum.* **a** Workflow for preparing lysate for in vitro translation assay. **b** Plasmid construct to generate mRNA transcripts containing a *P. falciparum* specific 5′ and 3′ UTR with a firefly luciferase open reading frame. Maxipreps of the plasmid were digested with PvuII and BamHI to create the templates for T7 transcription to make a final mRNA with the *P. falciparum* UTRs and firefly luciferase. **c** Lysates were incubated in the presence of a 10 × magnesium-containing translation buffer and luciferase mRNAs for a time course of 30 min to 120 min followed by the addition of luciferin reagent to assay for luciferase activity. **d** Lysates were incubated with DMSO control only or with 5 μM cycloheximide before or after the 120-min incubation followed by assaying for luciferase output

Building upon the work of Ferreras et al., an in vitro translation assay was further developed and optimized from *P. falciparum* cultures with the addition of exogenous firefly luciferase reporter mRNAs to allow for high-throughput plate-based luciferase assay screening (Fig. 1a) [12]. Cultures were scaled up to 500 ml HYPERFlasks (Corning) using synchronized, high-density, late trophozoite cultures, and utilized a saponin lysis method to release the parasites from the red blood cells (see “Methods” section). To maintain consistency of lysis conditions, a cell homogenizer (Isobiotec, Germany) was utilized, which gently and uniformly breaks open cells by passing the lysate though a precise 4-µM ball-bearing clearance, a technique that has found widespread use in sub-cellular fractionation studies [13]. Harvesting higher parasitaemia cultures in small lysis buffer volumes resulted in robust and reproducible translation activity when the A_280_ measured in the range of 7–20 mg/mL. Lysate competency for in vitro translation was measured by the translation of the firefly luciferase reporter constructs with a *P. falciparum* 5′ untranslated region (UTR) of the ubiquitously expressed erythrocyte binding antigen (EBA-175) and a 3′UTR from the *P. falciparum* histidine rich protein (*Pf* HRP) (Fig. 1b). These extracts achieved luciferase saturation similar to commercially available rabbit reticulocyte lysate (Retic Lysate IVT, Thermo Fisher Scientific), although with slower kinetics (Fig. 1c). To verify that the activity of the lysates did not result from the negligible amount of ribosome-containing reticulocytes potentially present in red blood cell (RBC) cultures, lysates were isolated from uninfected RBC cultures. To attain a comparable concentration of material (as measured by A_280_) from the uninfected RBCs (uRBCs) to the *P. falciparum* lysates, uRBCs were concentrated 10-fold using a Centricon YM-10 spin column (EMD Millipore, Hayward, CA, USA) and the in vitro translation assay was performed in an identical manner with a simultaneously prepared *P. falciparum* lysate in the presence or absence of magnesium (Additional file 1). Concentrated uRBCs were unable to produce luciferase activity, indicating that the magnesium-dependent translation activity in this assay is exclusively due to the presence of *P. falciparum* ribosomes and not from residual human ribosomes present in RBCs. Each batch of *P. falciparum* lysate varies in terms of maximum luciferase output and time to reach that maximum. Thus, for each harvest, a preliminary kinetic assay is necessary to establish the optimal incubation time (defined as ~80 % of maximum translational activity).

**Fig. 2:**
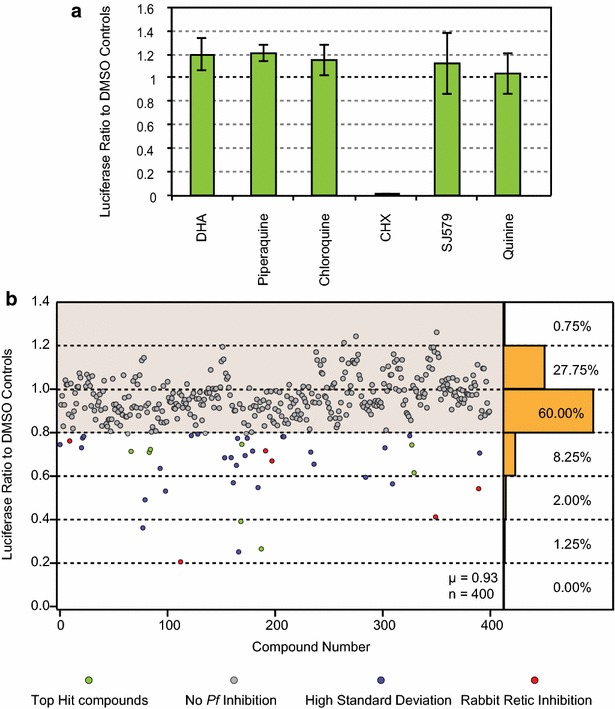
In vitro translation assay drug screens. **a** Lysates were incubated in the presence of anti-malarials and cycloheximide, a general eukaryotic translation inhibitor, all at 1 μM final concentration. *Error bars* represent the standard deviation among three biological replicates. **b** MMV Malaria Box compounds were added to lysates at a 1 μM final concentration. The average of three biological replicates were used to determine the extent of translation inhibition and normalized to the average of the DMSO controls present in each plate. Each point on the *graph* is the averaged response of a single drug. Each point is colour-coded by type of effect: top hit compound, no *P. falciparum* inhibition, high standard deviation, or inhibition in rabbit reticulocyte. The histogram on the *right* of the graph displays the total percentage of compounds with the given luciferase ratio

To verify that this assay specifically measures protein synthesis, translation activity of the lysate was assessed in the presence of 5 μM cycloheximide (27× reported IC_50_ = 181 nM) [14], an inhibitor of general eukaryotic translation elongation. When cycloheximide is added prior to the 37 °C incubation, translation is inhibited, resulting in the absence of luciferase expression, however, when added following the 37 °C incubation there was no effect on translation and the final luciferase activity was comparable to a control DMSO sample (Fig. 1d). Furthermore, to confirm that the assay is specific for inhibitors of protein synthesis, and not general anti-malarial activity, several anti-malarial compounds at 1-μM concentration (dihydroartemisinin (DHA), piperaquine, chloroquine, SJ579, and quinine) were tested along with 1-μM cycloheximide and found that none significantly inhibited translation in the assay with the exception of cycloheximide (Fig. 2a). Additionally, in both the 1-μM and 5 μM cycloheximide concentrations, in every lysate assayed, the measured luciferase activity was equivalent to the background (see Fig. 1d and Additional file 1).

### Screening the MMV Malaria Box for translational inhibitors

**Fig. 3:**
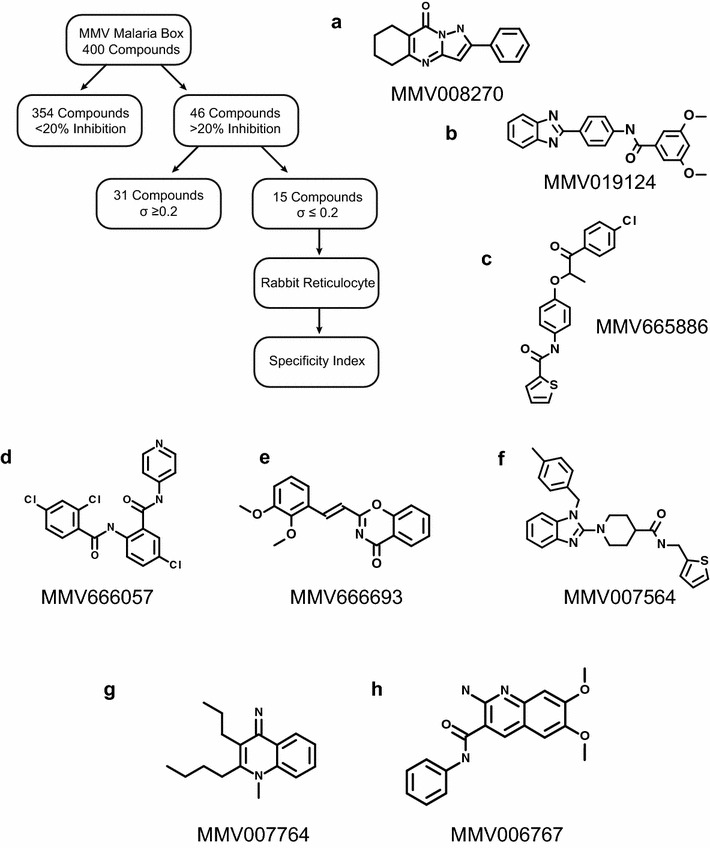
Flow diagram and results of the Malaria Box screen. Starting with 400 compounds, each compound was tested in three independent biological replicates in in vitro translation assays with 1 μM of the compound. Of the 46 compounds that achieved at least 20 % inhibition of translation, only eight passed both the standard deviation filter, and specificity filter removing general eukaryotic translation inhibitors (as measured by translation inhibition in rabbit reticulocyte lysate) and luciferase inhibitors. **a**–**h** Structures of the eight compounds that passed the primary and secondary screens. The letter of each structure is matched to the compounds listed in Table 1

**Fig. 4:**
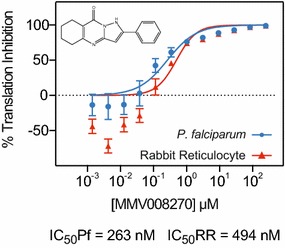
Dose–response *curves* of MMV008270. Dose-response *curves* in *P. falciparum* lysate or rabbit reticulocyte lysate using a 12-point 1:3 titration starting at 250 μM. Data were normalized to background and DMSO-only controls. IC_50_ values for each system are listed below the *graph*

Screening of the Malaria Box was performed using a 1-µM drug concentration for 1.5 h at 37 °C using the existing layout of the Malaria Box. Each plate was replicated at least three times and normalized to the average of all the control wells situated at the peripheral columns of each plate, totaling 16 controls wells per plate. A stop solution of 5 μM cycloheximide was added immediately following the incubation to simultaneously stop all translation in all wells, providing for uniform temporal measurements of translation throughout the luminescence assay. The results of this screen were calculated as a fractional value of the maximum in vitro translation normalized to the average of DMSO controls (Fig. 2b). Standard high throughput screen assay parameters were calculated (Additional file 2) and resulted in a signal-to-noise ratio of >3000, a coefficient of variation of 20.12 % and a Z’ factor of 0.87. Of all 400 wells assayed, 88.5 % (n = 354) of the compounds showed little to no inhibitory effect on translation (normalized luciferase ratio >0.80) whereas 46 compounds showed at least a 20 % inhibition of luciferase activity. To be considered further, a conservative cut-off was imposed for the standard deviation between replicates to be less than 0.20 of the normalized luciferase ratio of each sample. Although the Malaria Box was previously screened for cytotoxic effects, a secondary screen was performed on the final 15 compounds (standard deviation of <0.20 and >20 % normalized luciferase inhibition) using commercially available rabbit reticulocyte lysate (also in 1 μM drug concentration with two replicates) to ensure that positive hits did not also block mammalian in vitro translation or inhibit luciferase activity (Retic IVT, Life Technologies). A specificity index was defined as the ratio of normalized rabbit reticulocyte average translation over the normalized *P. falciparum* average translation. Specificity ratios ranged from 0.42 to 2.14, with values >1 signifying compounds that have a greater inhibitory effect on *P. falciparum* translation than rabbit reticulocyte translation, a proxy for general mammalian translation. After filtering for *P. falciparum* inhibition, standard deviation, rabbit reticulocyte inhibition, and large specificity ratios, eight compounds were identified for further analysis (Fig. 3). These eight compounds showed a ratio of >1.2 and are promising hits for further optimization and lead development. The top hit compound, MMV008270, had a specificity ratio of 1.96 and inhibited *P. falciparum* in vitro translation by >70 % on average when assayed at 1uM drug concentration. Next, dose–response curves of MMV008270 were characterized to calculate the half-maximal inhibitory concentration (IC_50_) value in both the *P. falciparum* and rabbit reticulocyte systems. Dose–response assays were performed in duplicate, with a 12-point 1:3 dilution series starting at a high concentration of 250 μM (Fig. 4). The resulting IC_50_ value for *P. falciparum* was 263 nM whereas the IC_50_ value for rabbit reticulocyte was 494 nM, a 1.89× higher IC_50_ value and consistent with the initial specificity index of 1.96× higher translation inhibition from the initial Malaria Box screen. The CHEMBL MMV databases were inspected for bioassays results of this compound. A listed Novartis study on the cytotoxicity in human fibroblasts calculates an IC_50_ for this compound above 32 μM suggesting low in vivo activity or penetrance in mammalian cells (Table 1) [15]. Additionally, an analogue (MMV006937) of the hit compound included in the Malaria Box set differs by the presence of a single hydrogen bond on the benzene ring and did not inhibit translation in the in vitro assay (% normalized luciferase translation MMV006937 = 83 %), suggesting a high degree of structural specificity associated with the inhibitory activity of the original hit. Further structure activity relationship studies of this compound will focus on the development of more specific and potent inhibitors of *P. falciparum* translation relative to a mammalian counterpart.

**Table 1:** Eight top hit compounds

Chemical structure	CHEMBLID	MMV number	Alternative compound number	Average *Pf* IVT	Specificity index	CHEMBL IC50 in uM	CHEMBL IC50 in human fibroblast (MRC-5) cells to measure toxicity
a	CHEMBL600904	MMV008270	GNF-Pf-780	0.27	1.96	1.93	>32000 nM
b	CHEMBL536393	MMV019124	TCMDC-123658	0.39	2.05	0.51	>32000 nM
c	CHEMBL532525	MMV665886	TCMDC-125438	0.62	1.69	0.77	>32000 nM
d	CHEMBL589060	MMV666057	TCMDC-125853	0.71	1.55	0.61	>32000 nM
e	CHEMBL546994	MMV666693	TCMDC-124577	0.71	1.57	0.06	>32000 nM
f	CHEMBL588732	MMV007564	GNF-Pf-4877;TCMDC-124400	0.72	1.51	0.53; 0.75	>32000 nM
g	CHEMBL1198651	MMV007764	GNF-Pf-4338	0.74	1.59	0.31	=19329.43 nM
h	CHEMBL530149	MMV006767	GNF-Pf-3828;TCMDC-123992	0.75	1.61	0.41; 1.13	>32000 nM

After filtering for several attributes, this study offers a final list of eight hit compounds. The chemical structure letter corresponds to the structures found in Fig. 3. Listed in this Table are the CHEMBL names, MMV number, any alternative compound name found in databases, the average normalized luciferase output, specificity index, the CHEMBL IC_50_, and CHEMBL cytotoxicity IC_50_ data from human fibroblast (MRC-5) cells

## Discussion

This work presents a novel high-throughput luciferase assay that enables the discovery of compounds inhibiting eukaryotic protein synthesis in *P. falciparum*. This cell-free system is sensitive to the known translation inhibitor cycloheximide, whereas anti-malarials with no known effect on translation, such as chloroquine and DHA, do not exhibit inhibitory activity in the assay. Utilizing this novel assay resulted in the identification of eight compounds that are inhibitors of *P. falciparum* translation, and more effectively inhibit *P. falciparum* than mammalian in vitro translation. Considering that this assay is based on a crude extract that naturally has more variability than a pure enzyme assay, additional secondary screening (such as IC curves in this case) is required to establish their validity. Of these eight compounds, further characterization of the top hit, MMV008270, showed 1.9× higher activity against *P. falciparum* translation over a general eukaryotic translation system. While a 1.9× difference is likely to be too narrow for serious consideration as a lead molecule, it provides a starting point for further structure activity relationship studies with the goal of widening the gap between *P. falciparum* and mammalian inhibition. Further, target validation by selection for resistance may aid in determining which component of the ribosomal machinery is the target of this compound. It is likely that multiple components of the ribosome could be targeted simultaneously to yield a synergistic effect.

The assay developed here has the potential to be a powerful tool for additional translation-specific drug screens as well as answering important questions of basic *Plasmodium* biology. For example, future experiments using this streamlined, multi-well, plate-friendly assay will allow for the further investigation of *cis*-acting determinants of translational efficiency that were identified in a previous study [16]. In total, this work presents a valuable molecular tool for probing an essential process in *P. falciparum* biology.

## Availability of supporting data

The data set supporting the results of this article will be freely available in the CHEMBL MMV repository (https://www.ebi.ac.uk/chembl/malaria/).

## Additional files

10.1186/s12936-016-1231-8 Luciferase expression is a product of *Plasmodium falciparum* magnesium-dependent translation. Comparison of luciferase expression in *P. falciparum* lysate and in concentrated red blood cell lysate harvested in the same manner as *P. falciparum* lysate.
10.1186/s12936-016-1231-8 Standard high throughput assay parameters. A. Seven different lysates were assayed in the presence or absence of the luciferase reporter mRNA to calculate the signal-to-noise ratio. B. A histogram showing all DMSO controls from the screen. The percent coefficient of variation was calculated from these controls. C. Calculating the Z’ factor from a single lysate that was assayed in the presence or absence of the luciferase reporter mRNA using either the raw or normalized controls. For this calculation, the positive controls are in the absence of mRNA and the negative controls contain luciferase mRNA.
