# Supplementary figures and images for: Identification of Plasmodium falciparum specific translation inhibitors from the MMV Malaria Box using a high throughput in vitro translation screen
Source: Malar J. 2016 Mar 17;15:173. doi: 10.1186/s12936-016-1231-8 (PMC4794828; doi:10.1186/s12936-016-1231-8)

Figure S1

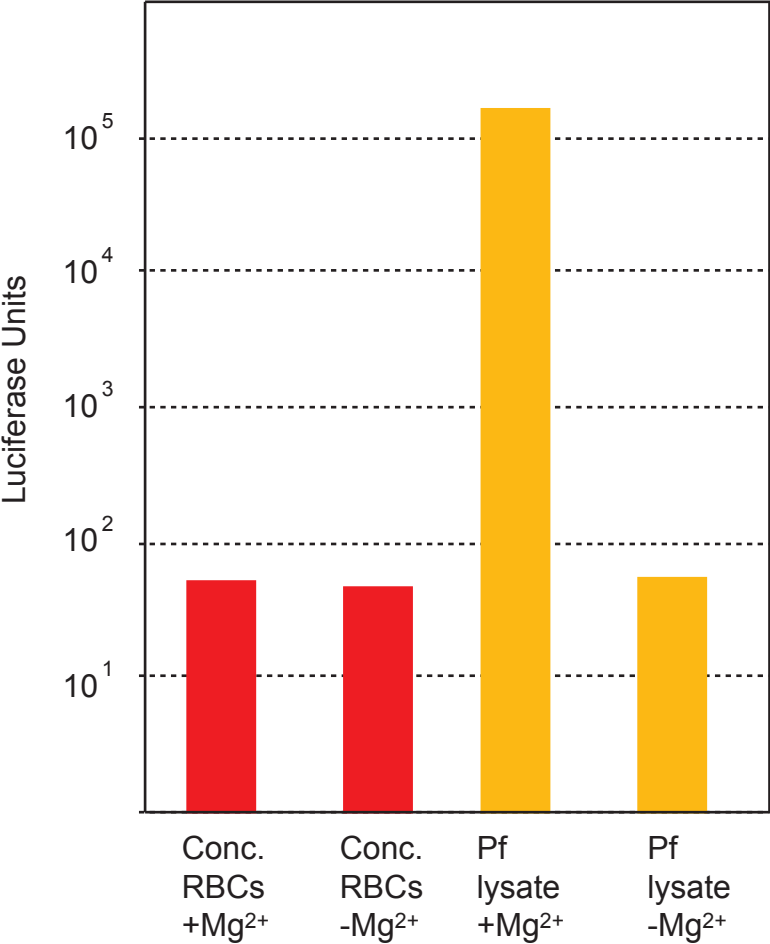

Supplement: Supplementary file 1 — 10.1186/s12936-016-1231-8 Luciferase expression is a product of Plasmodium falciparum magnesium-dependent translation. Comparison of luciferase expression in P. falciparum lysate and in concentrated red blood cell lysate harvested in the same manner as P. falciparum lysate. [file 12936_2016_1231_MOESM1_ESM.pdf]

Figure S2

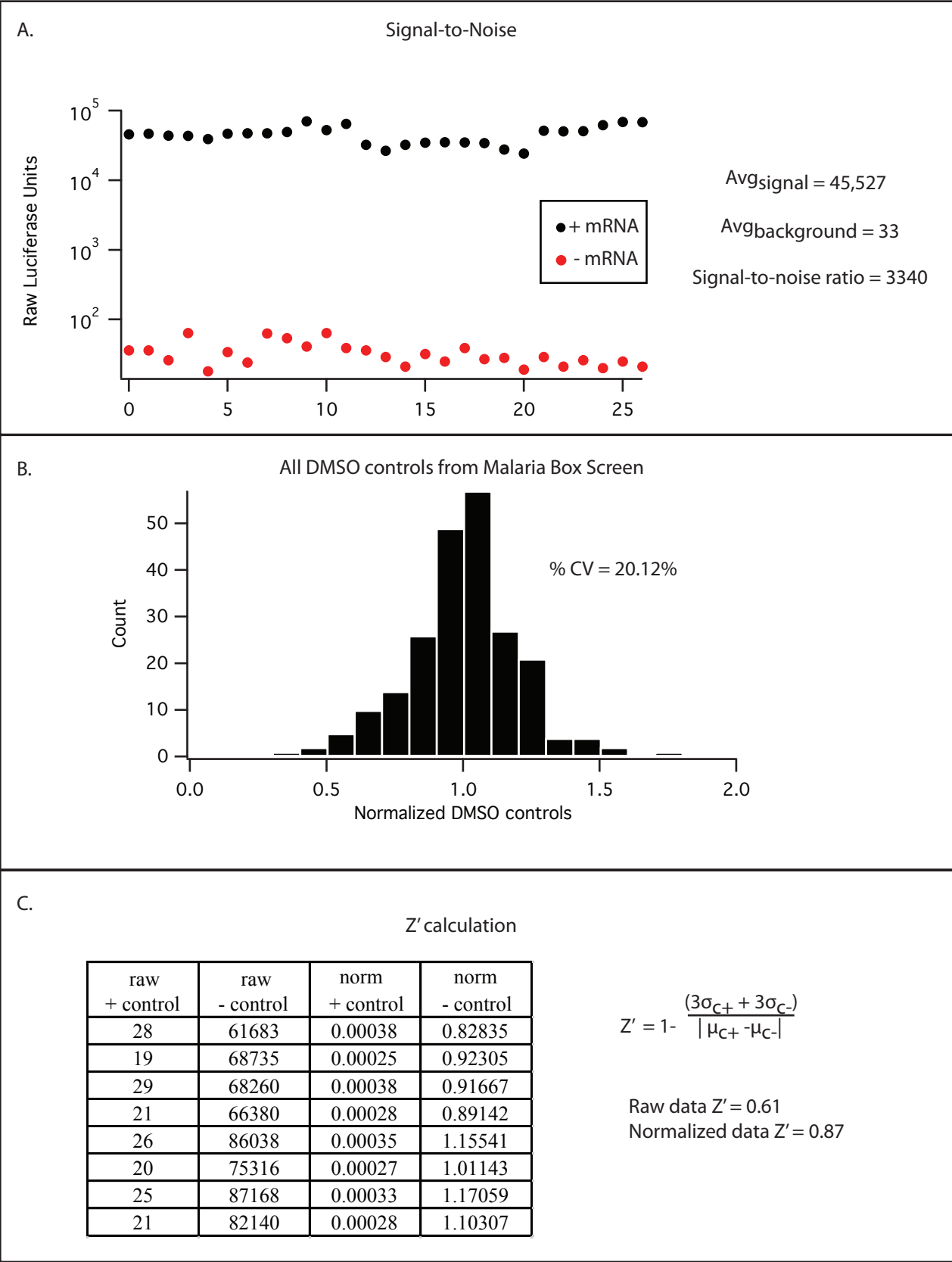

Supplement: Supplementary file 2 — 10.1186/s12936-016-1231-8 Standard high throughput assay parameters. A. Seven different lysates were assayed in the presence or absence of the luciferase reporter mRNA to calculate the signal-to-noise ratio. B. A histogram showing all DMSO controls from the screen. The percent coefficient of variation was calculated from these controls. C. Calculating the Z’ factor from a single lysate that was assayed in the presence or absence of the luciferase reporter mRNA using either the raw or normalized controls. For this calculation, the positive controls are in the absence of mRNA and the negative controls contain luciferase mRNA. [file 12936_2016_1231_MOESM2_ESM.pdf]
